# Intravenous injections of the oncolytic virus M1 as a novel therapy for muscle-invasive bladder cancer

**DOI:** 10.1038/s41419-018-0325-3

**Published:** 2018-02-15

**Authors:** Cheng Hu, Ying Liu, Yuan Lin, Jian-Kai Liang, Wen-Wen Zhong, Ke Li, Wen-Tao Huang, De-Juan Wang, Guang-Mei Yan, Wen-Bo Zhu, Jian-Guang Qiu, Xin Gao

**Affiliations:** 1 Department of Urology, The Third Affiliated Hospital of Sun Yat-sen University, Guangzhou, Guangdong China; 2 Department of Infectious Diseases, The Third Affiliated Hospital of Sun Yat-sen University, Guangzhou, Guangdong China; 3 Department of Pharmacology, Sun Yat-sen University, Guangzhou, China; 4 Department of Urology, The Sixth Affiliated Hospital of Sun Yat-sen University, Guangzhou, Guangdong China; 5 Collaborative Innovation Center for Cancer Medicine, Guangzhou, China

## Abstract

Muscle-invasive bladder cancer (MIBC) is associated with low survival and high recurrence rates even in cases in which patients receive systemic treatments, such as surgery and chemotherapy. Here, we found that a naturally existing alphavirus, namely, M1, selectively kills bladder cancer cells but not normal cells, findings supported by our observations of changes in viral replication and MIBC and patient-derived MIBC cell apoptosis. Transcriptome analysis revealed that interferon-stimulated genes (ISGs) are expressed at low levels in sensitive bladder cancer cells and high levels in resistant cells. Knocking down ZC3HAV1 (ZAP), an antiviral factor in ISGs, restores M1 virus reactivity in resistant cells, and overexpressing ZAP partially reverses M1 virus-induced decreases in cell viability in sensitive cells. In orthotopic MIBC mice, tail vein injections of M1 significant inhibit tumor growth and prolong survival period, antitumor effects of M1 are stronger than those of the first-line chemotherapy agent cisplatin (CDDP). Treated tumors display enhanced cleaved-caspase-3 signals, which are representative of cell apoptosis, and decreased Ki-67 signals, which are representative of cell proliferation. Moreover, tissue microarray (TMA) analyses of clinical tumor specimens revealed that up to 45.6% of cases of MIBC presented with low ZAP expression, a finding that is prevalent in advanced MIBC. Our results indicate that the oncolytic virus M1 is a novel agent capable of functioning as a precise and effective therapy for MIBC.

## Introduction

Bladder cancer is the most common malignancy of the urinary system^1^, approximately one-quarter of bladder cancers are muscle-invasive bladder cancers (MIBCs)^2^, whose incidence and mortality are elevated in China^3^. More than 90% of MIBCs are transitional cell carcinomas^4^. Radical cystectomy and cisplatin (*cis*-diamminedichloridoplatinum (CDDP))-based chemotherapy remain the standard first-line treatments for MIBC. Radical cystectomy and urinary diversion facilitate total removal of the primary tumor, however, affected patients must subsequently endure multiple reconstructive surgeries, and the 5-year survival rate among patients receiving these treatments is only 40–60%^5^. Cisplatin-based chemotherapy has been reported to yield a 6-year progression-free survival rate of 3.7%^6^, however, this regimen is highly toxic to patients and is even associated with a mortality rate of approximately 4%^7^. Comprehensive assessments of the benefits, risks, and side effects of cisplatin-based chemotherapy indicate that this therapy is not appropriate for part of post-surgery patients or patients with end-stage disease^8^. Despite the efforts of clinicians and researchers in past decades, survival among patients with MIBC has not improved^9–11^. New approaches to the treatment of MIBC are being continuously investigated to facilitate the development of treatments with superior efficacy and lower toxicity.

Oncolytic viruses are genetically engineered or naturally existing viruses that can selectively infect, replicate in, and lyse cancer cells while exerting minimal harmful effects on normal cells^12^. Because they have the capability to target and kill tumors and induce antitumor immunity, oncolytic virotherapies appear to be promising oncologic therapeutic agents^13–15^. Our previous study showed for the first time that alphavirus M1, a Getah-like viral strain isolated in China^16,17^, is an oncolytic virus^16,18^. A safety study showed that administering 18 separate intravenous doses of M1 (1×10^9^ plaque-forming unit (PFU) per dose) to cynomolgus macaques induced no toxicity^19^. Such a safe and potent oncolytic virus seems to be a good choice as a treatment for patients with MIBC.

In this study, we sought to test the possible benefits of M1 oncolytic therapy in MIBC. We confirmed that ZAP protein plays an important role in determining the sensitivity of MIBC cells to M1 and found that ZAP was expressed at low levels in 45.6% of bladder cancer samples. These data serve as basic evidence indicating that M1 oncolytic therapy may be useful for the treatment of MIBC.

## Results

### M1 selectively kills bladder cancer cells but not normal cells

**Fig. 1:**
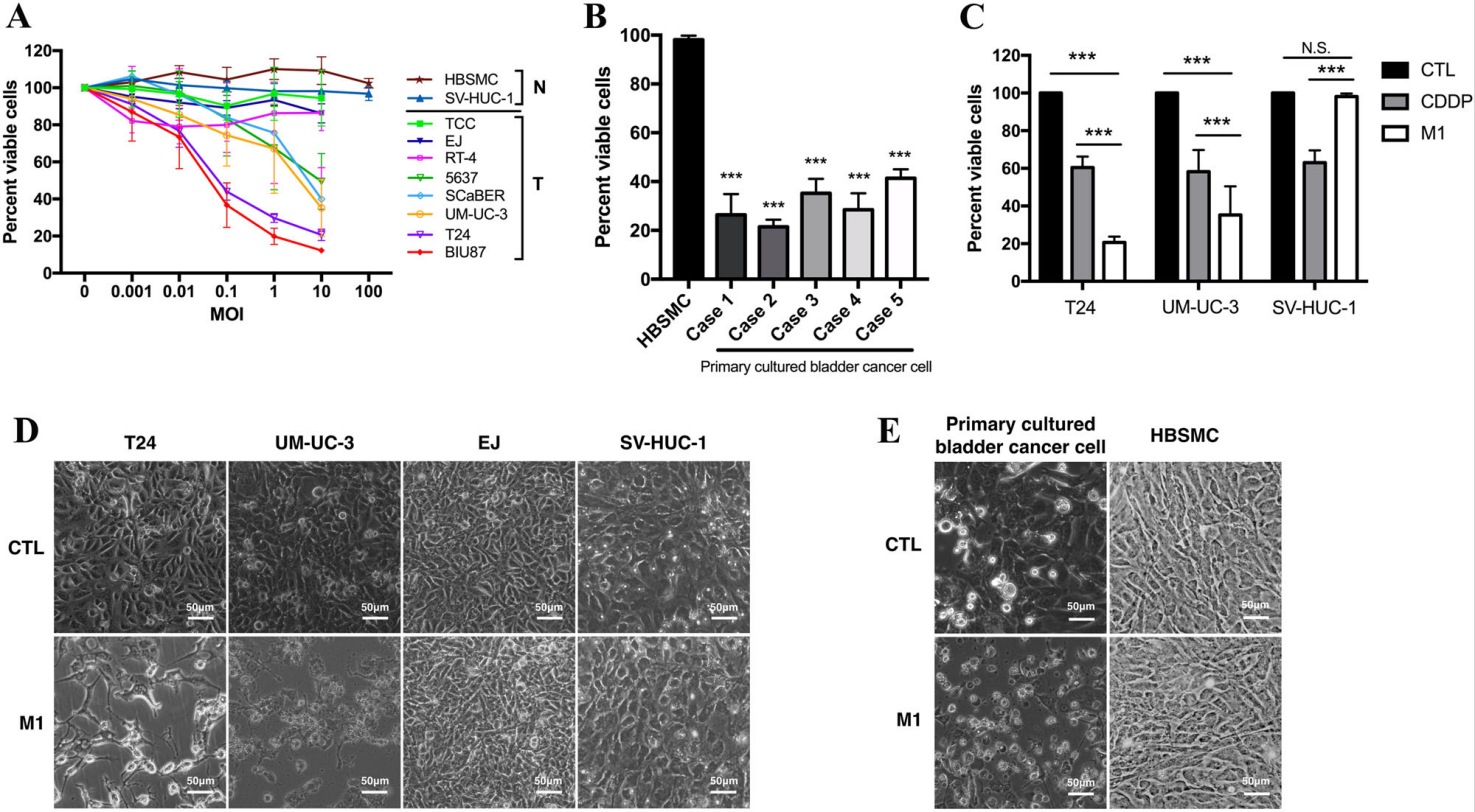
Effect of M1 on bladder cancer cell viability. **a** Evaluations of the viability of several bladder tumor (T) and normal (N) cell lines were performed by MTT assay after the cells were exposed to M1. Each color represents one cell line. **b** Five primary cultured bladder cancer tissue specimens were isolated from a clinical bladder cancer tumor specimen, and the responsiveness of each specimen to M1 was assessed and compared with that of a primary normal bladder tissue specimen (HBSMCs). **c** The bladder cancer cell lines T24 and UM-UC-3 and the normal cell line SV-HUC-1 were treated with CDDP (10 μM) and M1 (MOI = 10 PFU per cell), and cell viability assay was performed at 48 h post-infection. **d,** **e** Observation of the morphology of various bladder cells exposed to M1 (MOI = 10 PFU per cell) for 48 h. **d** Bladder cancer cells (T24, UM-UC-3, and EJ) and normal cells (SV-HUC-1), **e** Primary cultured bladder cancer cells and normal cells (HBSMCs). ***p < 0.001; N.S., not significant

To assess the oncolytic efficacy of M1, we initially detected the viability of eight bladder cancer cell lines (T24, BIU87, UM-UC-3, SCaBER, 5637, RT-4, EJ, and TCC). We found that M1 significantly decreased cell viability to varying degrees in most of the cell lines (Figs. 1a, d). We classified the cells into highly, moderately lowly sensitive cells, and normal cells according to the viability of the cells following M1 exposure (Fig. S1). To test the basic safety of M1 oncolytic therapy, we measured the effects of M1 on survival in the normal bladder cell lines HBSMC and SV-HUC-1. As shown in Fig. 1a, the multiplicity of infection (MOI) = 1 plaque-forming unit per cell (pfu/cell) of M1 ranged from 0.001 to 100, indicating that the virus had no harmful effects on normal cells. Furthermore, we assessed the effects of M1 on five cases of patient-derived bladder cancer. Consistent with the above results, these results showed that M1 (MOI = 10) killed >50% of the patient-derived cells but did not harm the HBSMCs (Figs. 1b, e).

We also compared the antitumor effects of M1 on two bladder cancer cell lines (T24 and UM-UC-3) and a normal cell line (SV-HUC-1) with those of conventional chemotherapy drugs, we found that the cell viability was lower after the treatment of M1 in bladder cancer T24 and UM-UC-3 cells, higher in normal bladder cell SV-HUC-1 compared with cisplatin (Fig. 1c).

### M1 induces apoptosis in bladder cancer cells

**Fig. 2:**
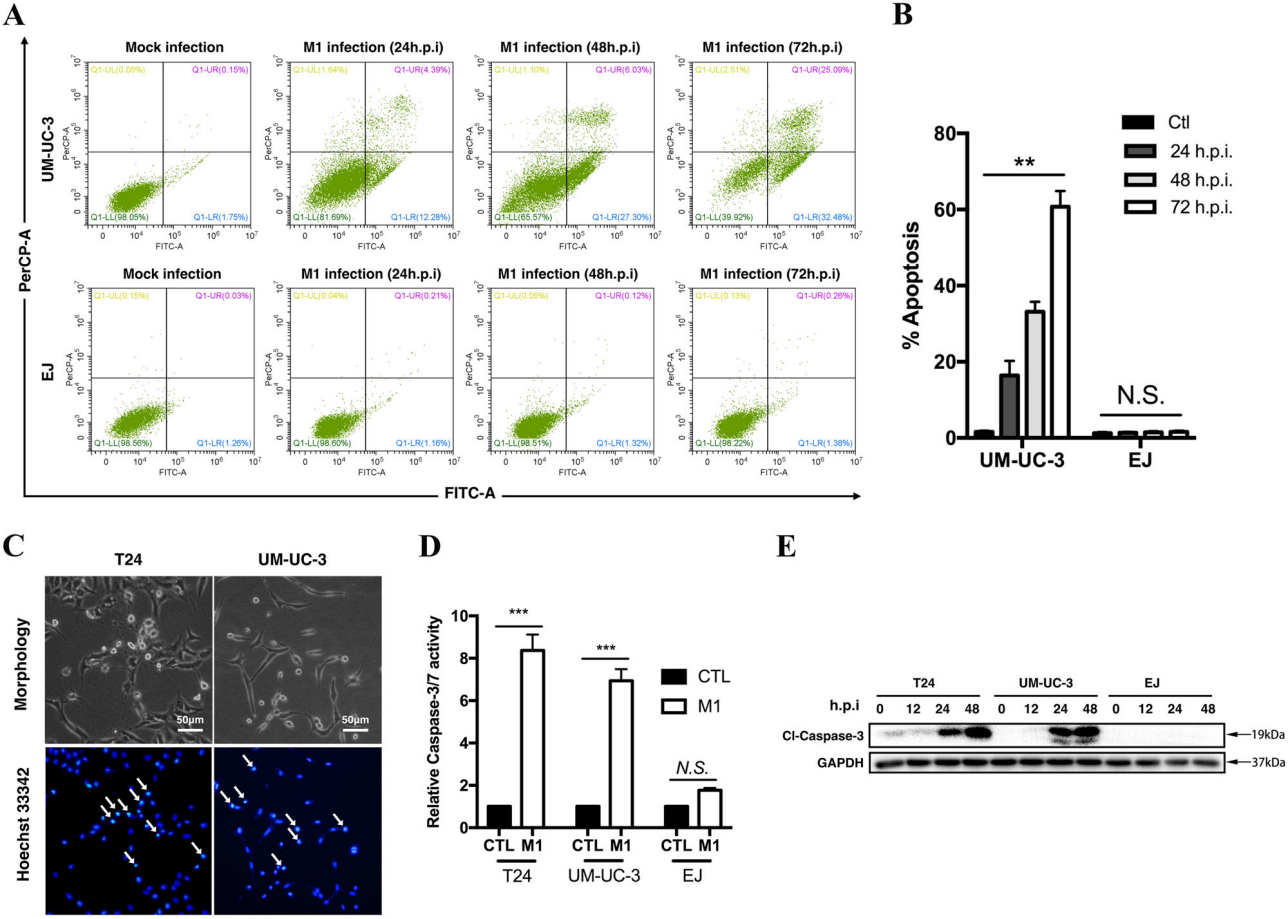
M1 increases apoptosis in susceptible bladder cancer cells. **a** Flow cytometry and annexin V staining analyses of UM-UC-3 and EJ cells treated with M1 (MOI = 1 PFU per cell) and mock control agents. **b** Numbers of apoptotic cells, as determined by flow cytometry at 24, 48, and 72 h post-infection. **c** Chromatin condensation was demonstrated via Hoechst 33342 staining. T24 and UM-UC-3 cells were treated with M1 (MOI = 10 PFU per cell) for 72 h. Chromatin condensation is indicated by the white arrows. **d** Caspase-3/7 activity in T24, UM-UC-3, and EJ cells treated with M1 (MOI = 10 PFU per cell) for 48 h. **e** Western blots was performed to detect caspase-3 protein expression in the indicated cell lines at 0, 12, 24, and 48 h after M1 (MOI = 10) infection. GAPDH glyceraldehyde-3-phosphate dehydrogenase, CTL control; hpi, hours post-infection. **p < 0.01; ***p < 0.001; N.S., not significant

We next determined the type of cell death induced by M1. Flow cytometry showed that the proportions of apoptotic cells increased in a time-dependent manner (Fig. 2a). At 48 and 72 h after M1 infection, the number of cells undergoing apoptosis had significantly increased in the corresponding group compared with the control group (Fig. 2b). We confirmed that cell apoptosis had occurred by performing Hoechst staining, which demonstrated that chromatin condensation had occurred in response to M1 treatment. Transmission electron microscopy images found the size of endoplasmic reticulum was correlated with the sensitivity of cells to M1 virus (Fig. S2). We also observed that activated caspase-3 and cleaved-caspase-3 levels were increased at 48 h post-treatment in the corresponding group compared with the control group (Figs. 2c-e). These results indicated that M1-induced apoptosis in sensitive bladder cancer cells.

### M1 replicates rapidly after infecting sensitive cancer cells but not resistant cancer cells

**Fig. 3:**
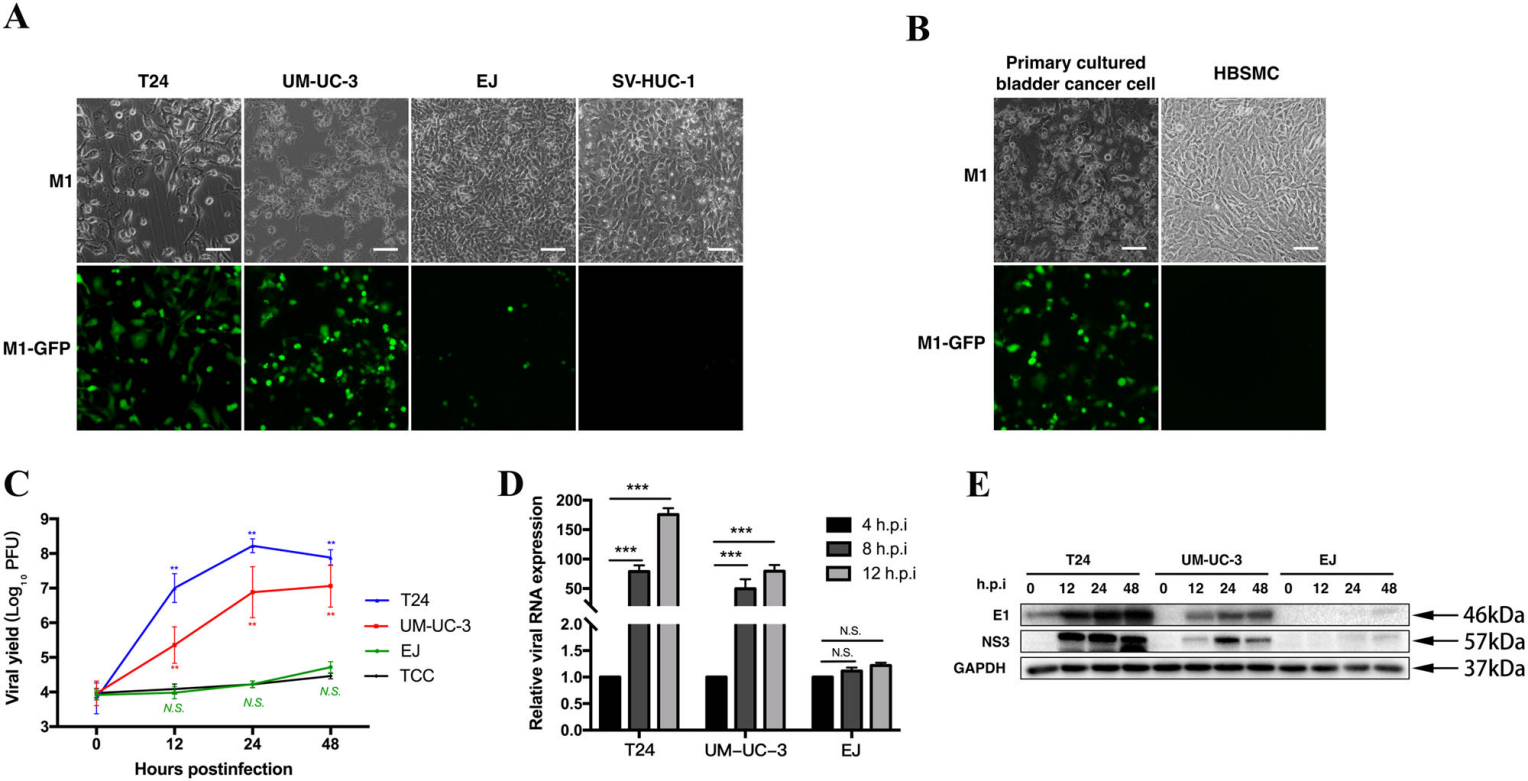
Viral replication induces apoptosis in susceptible cancer cells. **a**, **b** Various bladder cancer cells were infected with GFP fluorescently labeled M1 (MOI = 10) for 48 h, after which cell morphology and virus fluorescence were photographed. **a** Bladder cancer cell lines and normal cells. **b** Primary cultured bladder cancer cells and primary normal cells. **c** Time course of the viral titers in four representative cell lines after M1 infection (MOI = 10). Each color represents one cell line. **d** The indicated cell lines were infected with M1 (MOI = 10), and ZAP mRNA expression was quantified by qRT-PCR at 4, 8, and 12 h post-infection. Relative gene expression levels were normalized to β-actin expression levels. **e** Western blots showing the expression of the viral proteins E1 and NS3 in the indicated cell lines at 0, 12, 24, and 48 h after M1 infection (MOI = 10 PFU). GAPDH glyceraldehyde-3-phosphate dehydrogenase. **p < 0.01; ***p < 0.001; N.S, not significant

To determine the replicative state of M1, we established an engineered M1 labeled with green fluorescence protein (GFP). In the corresponding assay, high fluorescence intensity was representative of high viral replication levels. After 48 h of M1 infection, we detected strong GFP signals from the viruses in most of the sensitive cell lines (T24 and UM-UC-3) and the primary patient-derived bladder cancer cell line, indicating that M1 had replicated in those cell lines (Figs. 3a, b). However, only a limited number of signals were detected in refractory EJ cells and normal cells (SV-HUC-1 and HBSMC) (Figs. 3a, b). By measuring the viral yield over time, we found that M1 replicated rapidly in sensitive cell lines but not in the EJ and TCC cell lines (Fig. 3c), results consistent with those of the above fluorescence and cell viability experiments. Furthermore, we evaluated viral RNA and protein expression levels after M1 infection. As shown in Figs. 3d, e, viral RNA and protein significantly increased after infection in the T24 and UM-UC-3 cell lines but not in non-sensitive cells. Taken together, these findings indicate that rapid M1 replication occurs in sensitive cells, a phenomenon that is closely linked with M1-induced cell apoptosis.

### ZAP deficiency partly contributes to M1 viral replication in sensitive cancer cells

**Fig. 4:**
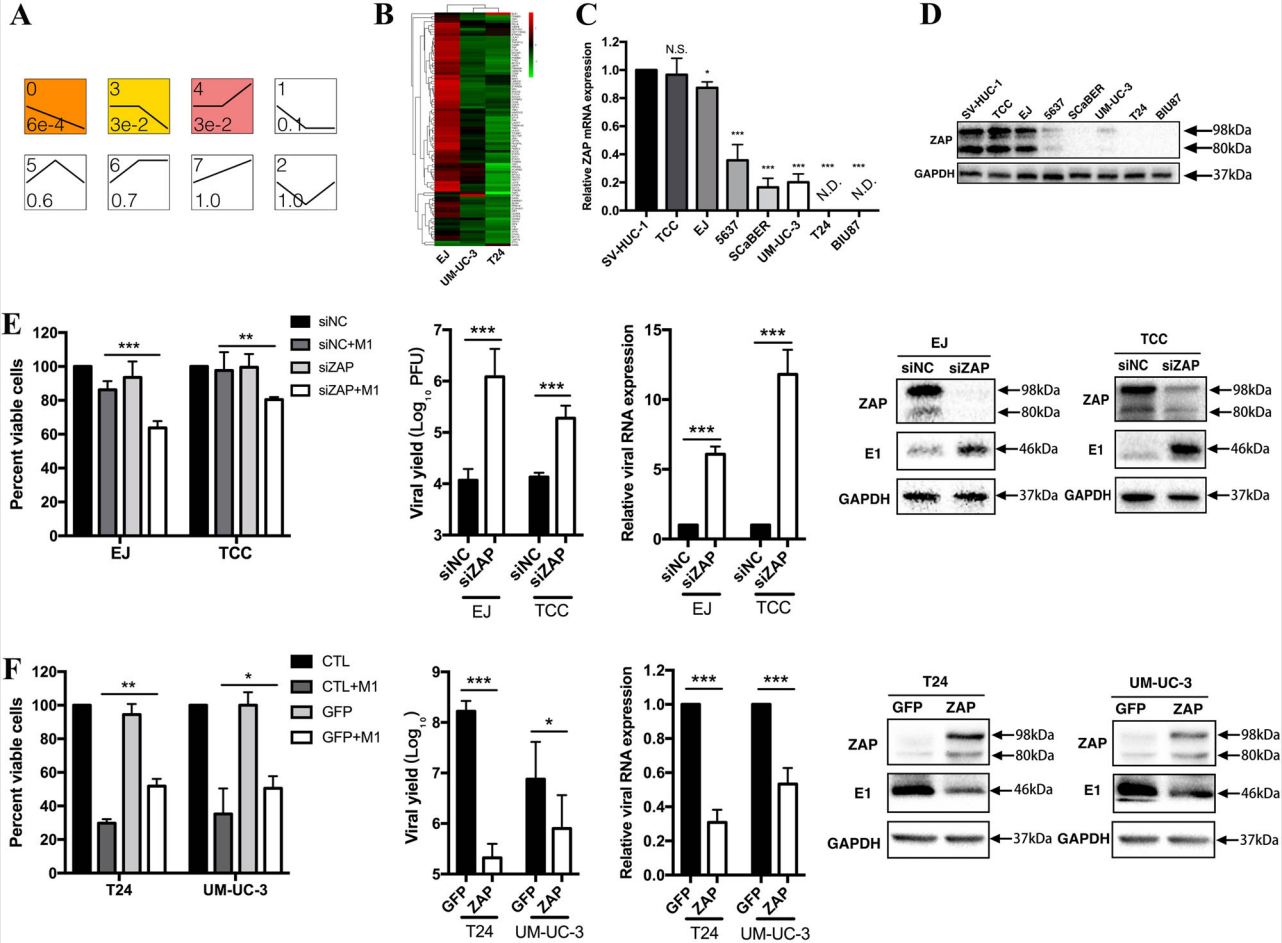
M1 sensitivity requires ZAP deficiency. **a** Trend clustering of the transcriptome profiles of the bladder cancer cell lines EJ, UM-UC-3, and T24. The profiles were ordered based on the significance of the differences in the number of genes assigned versus expected. Profile 0 consisted of genes whose expression was downregulated and significantly downregulated in three cell lines. The antiviral gene was in this profile. The number located in the upper-left corner is the profile serial number, and the number located in the lower-left corner is the *p*-value. **b** An analysis of the transcriptome data pertaining to the enrichment of the antiviral gene set was performed by evaluating gene expression levels in three different bladder cancer cell lines (EJ, UM-UC-3, and T24). Hierarchical clustering analysis was performed, and the genes expressed most significantly in the three cell lines were listed. **c** ZAP mRNA expression levels were detected in eight bladder cell lines and normalized to β-actin mRNA expression levels. **d** ZAP protein expression levels of the same eight cell lines were measured. GAPDH was used as a loading control. **e** ZAP was silenced in two representative resistant cancer cell lines (EJ and TCC) with siNC (negative control) and siZAP, and then the cells were infected with M1 (MOI = 10 PFU) for 48 h. Cell viability was detected by MTT assay, viral replication levels were determined by TCID_50_ essay, viral RNA expression was quantified by qRT-PCR, and viral protein expression was analyzed by western blotting. **f** ZAP was overexpressed in two representative sensitive cancer cell lines (T24 and UM-UC-3) with GFP (negative control) and ZAP, and then the cells were treated with M1 (MOI = 10 PFU) for 48 h. Cell viability, viral yield, and viral RNA and protein expression were evaluated as described above. Bar charts show the mean ± SD of three independent experiments. **e**, **f** All groups were compared with their respective control groups. siNC negative-control siRNA, TCID_50_ median tissue culture infective dose. *p < 0.05; **p < 0.01; ***p < 0.001; N.S, not significant

We aimed to elucidate the mechanism underlying M1 viral replication in sensitive cells. Given that virus replication is closely related to cell apoptosis, it is logical to conclude that antiviral immunity differs among cell lines, leading to differences in the responses of cells to M1. To test this hypothesis, we conducted transcriptome analysis involving highly sensitive T24 cells, moderately sensitive UM-UC-3 cells and resistant EJ cells, and then analyzed the expression levels of interferon (IFN)-stimulated genes (ISGs), which are crucial antiviral effectors that act against the viruses listed in the indicated public database (http://interferome.its.monash.edu.au/interferome/; http://lerner.ccf.org/labs/williams). We found that the ISG expression pattern in EJ cells was different from those in T24 and UM-UC-3 cells, as most ISGs were expressed at higher levels in the former cell line than in the latter two cell lines (Fig. 4b). These data strongly indicate that a weak antiviral signal in sensitive cells is an indicator that the cells are highly responsive to M1.

ZAP is an important gene that has previously been shown to be linked to M1 sensitivity in hepatoma cells^18^. Here, we evaluated whether ZAP also explains the sensitivity of bladder cancer cells to M1. We first examined ZAP expression levels in viable cell lines. We found that both ZAP mRNA and ZAP protein expression levels were correlated with the extent to which specific cell lines were sensitive to M1(Figs. 4c, d).

To determine the role of ZAP in the sensitivity of bladder cancer cells to M1 therapy, we conducted gain- and loss-of-function assays. We found that knocking down ZAP in EJ, TCC and normal cells (SV-HUC-1) significantly elevated the viral yield and the killing efficacy of M1(Fig. 4e and Fig. S3). Conversely, overexpressing ZAP in T24 and UM-UC-3 cells attenuated M1 viral replication and killing efficacy to a certain extent (Fig. 4f).

Collectively, these findings indicate that ZAP expression levels are negatively correlated with the replication and oncolytic effects of M1, suggesting that ZAP may be useful as a biomarker with which the sensitivity of bladder cancers to M1 therapy can be assessed in affected patients. As ZAP is one of the antiviral protein, which suggests that more ISGs may be involved in M1-induced immune system activity.

### M1 significantly represses orthotopic invasive bladder tumors

**Fig. 5:**
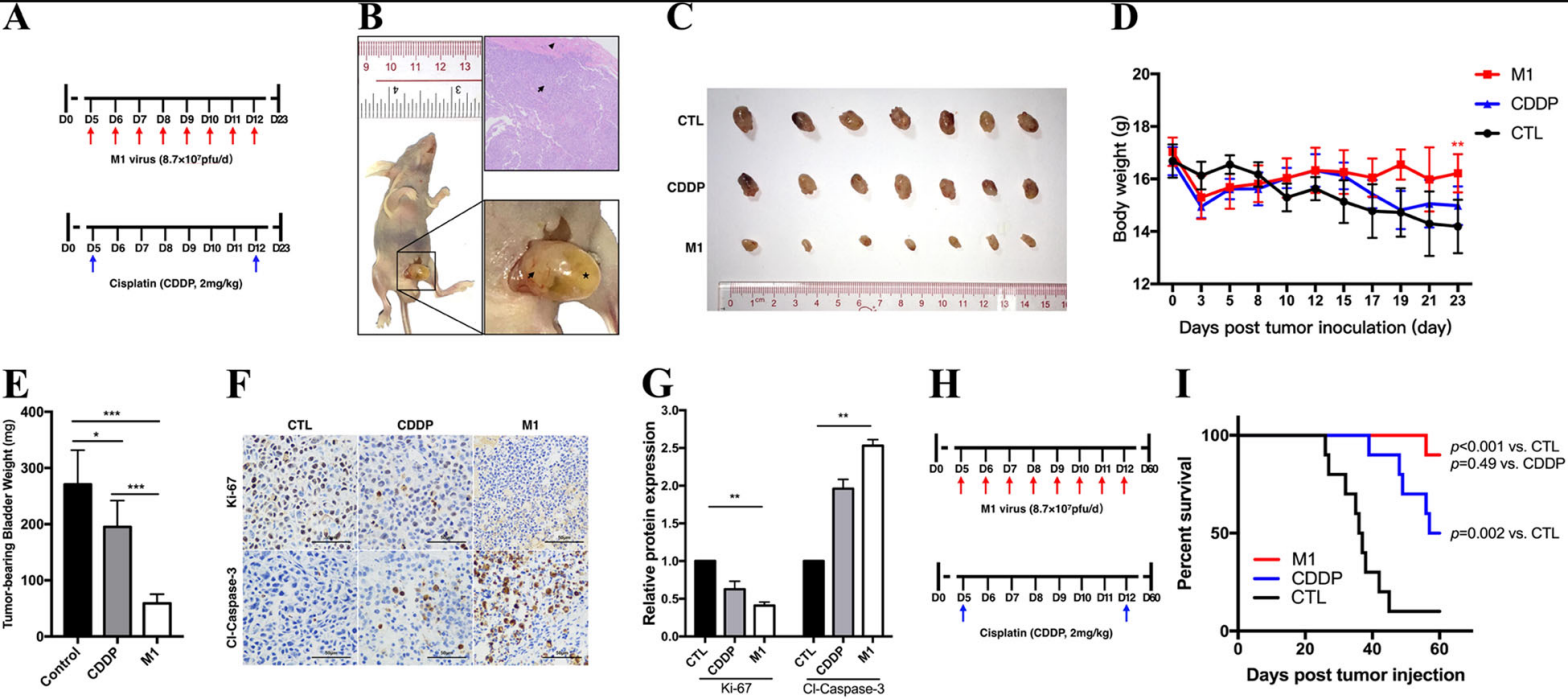
M1 significantly reduces orthotopic bladder tumor sizes. Establishment of the orthotopic bladder cancer mouse model. Laparotomy and bladder wall injections was used to establish the animal model. **a** We divided the animals into control, CDDP and M1 group (seven mice in each group), treatment in each group: CDDP, intraperitoneally at 2 mg/kg two times daily; M1, intravenously at 8.7 × 10^7^ PFU once daily for 8 days; and control, sterile PBS intravenously once daily for 8 days. i.v. intravenous injection, i.p. intraperitoneal injection. **b** Tumors visible to the naked eye formed 5–7 days after tumor inoculation. The image in the bottom-right corner is a high-magnification image of the image on the left. Arrow, bladder tumor; arrowhead, normal bladder smooth muscle; star, filled bladder cavity. **c** At the end of the experiment, all the mice were anesthetized and euthanized, and their tumor-bearing bladders were harvested and photographed. **d** The body weights of the tumor-bearing mice were recorded. **e** The weights of the tumor-bearing bladders were measured and recorded. **f** Immunohistochemistry was performed to detect Ki-67 and cleaved-caspase-3 expression. **g** Ki-67 and cleaved-caspase-3 expression levels in treated cells were compared with those in control cells, Relative protein expression levels were quantified with Image-Pro Plus 6.0 (IPP 6.0, Media Cybernetics, Rockville, MD). CTL control. **h** Timeline of experiment setup for **i**. Kaplan–Meier survival curve of mice bearing UM-UC-3 orthotopic tumors treated with PBS, CDDP, and M1 virus. Log‐rank with Holm–Sidak multiple comparison. *p < 0.05; **p < 0.01; ***p < 0.001

To evaluate the antitumor effects of M1 in vivo, we used UM-UC-3 cells to establish orthotopic bladder cancer models in female BALB/c-nu/nu mice. We divided the animals into three different groups (control, CDDP, and M1 groups, seven mice in each group) (Fig. 5a). As shown in Fig. 5b, successful establishment of the orthotopic-invasive bladder cancer model was confirmed by the immunohistochemistry staining results and our observation of bladder enlargement. M1 therapy significantly repressed UM-UC-3-derived orthotopic bladder tumor growth compared with CDDP therapy. It is worth noting that intravenous M1 administration did not affect the weights of the mice bearing tumors (Figs. 5c-e). Immunohistochemistry demonstrated that the tumor slices in M1 group displayed a significantly lower level of Ki-67, a marker of cell proliferation, and higher level of cleaved-caspase-3/7, a marker of cell apoptosis (Figs. 5f, g). Moreover, mice received M1 showed a statistically significant increase in overall survival (Figs. 5h, i). These results are consistent with those of the in vitro study, indicating that M1 is a potent intravenous drug.

### Relationship between tumor grade and ZAP deficiency

**Fig. 6:**
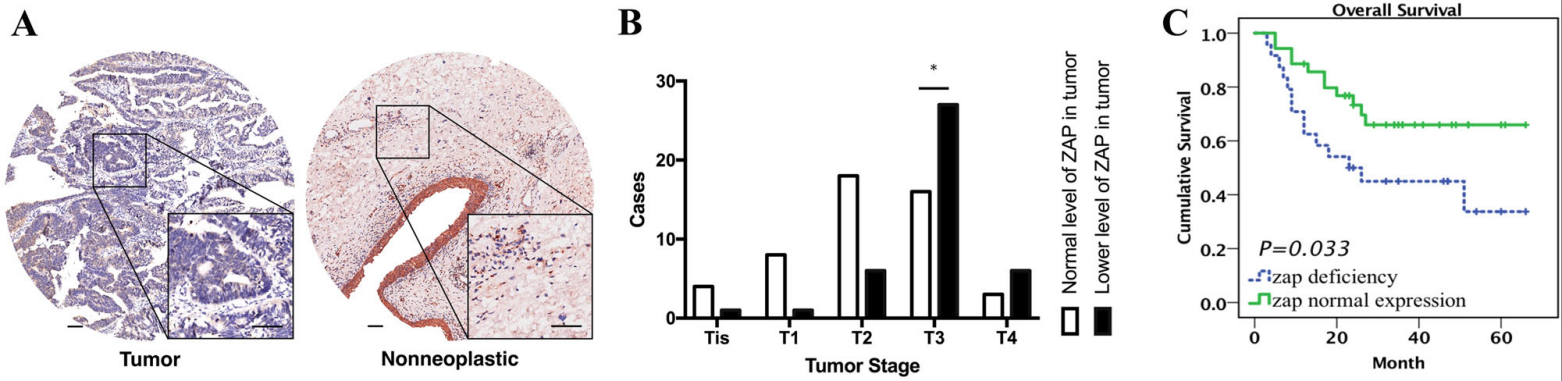
TMA analysis was performed to investigate the relationship between ZAP deficiency and tumor grade. **a** ZAP expression was detected by TMA analysis of clinical bladder cancer specimens. **b** Analysis of the relationship between ZAP expression and tumor stages revealed that ZAP deficiency was highly prevalent in advanced tumors. **c** Cumulative survival analysis indicated that ZAP deficiency was correlated with short overall survival

Our data show that ZAP expression is negatively correlated with the responsiveness of bladder cancer to M1. We sought to determine the exact proportion of bladder cancer patients with low ZAP protein expression levels, 91 pairs of bladder cancer tissue specimens and tumor/adjacent non-tumor tissue specimens were collected and TMA was performed (Table 1). ZAP expression was calculated in each tumor and adjacent non-neoplastic tissue specimen (Fig. 6a). A tumor-to-non-neoplastic tissue staining signal ratio <1 was indicative of low ZAP expression, which we observed in 45.6% of specimens. We also found that the incidence of low ZAP expression was significantly higher in tumors with higher grades (T3–4) than in tumors with lower grades (T1–2) (Fig. 6b). Moreover, we found that ZAP deficiency was correlated with poor cumulative survival durations (Fig. 6c). In summary, these results suggest that M1 has favorable effects on advanced bladder cancer.

**Table 1:** Clinicopathological characteristics of patient samples and expression of ZAP in bladder cancer patients and correlation between ZAP expression and Clinicopathological characteristics of bladder cancer patients

Characteristics		Total (*n*=90)	ZAP expression		Chi-square test *p*-value
			Low expression (45.6%)	Normal expression (54.4%)	
Gender	Female	14	5 (35.7%)	9 (64.3%)	0.421
	Male	76	36 (47.4%)	40 (52.6%)	
Age (years)	≤70	48	21 (43.8%)	27 (56.2%)	0.7131
	>70	42	20 (47.6%)	22 (52.4%)	
Anatomic stage	0is	5	1 (20.0%)	4 (80.0%)	0.0037
	I	6	1 (16.7%)	5 (83.3%)	
	II	24	5 (20.8%)	19 (79.2%)	
	III	37	24 (64.9%)	13 (35.1%)	
	IV	18	10 (55.6%)	8 (44.4%)	
T classification	Tis	5	1 (20.0%)	4 (80.0%)	0.0024
	T1	9	1 (11.1%)	8 (88.9%)	
	T2	24	6 (25.0%)	18 (75.0%)	
	T3	43	27 (62.8%)	16 (37.2%)	
	T4	9	6 (66.7%)	3 (33.3%)	
Node invasion	No	72	31 (43.1%)	41 (56.9%)	0.3409
	Yes	18	10 (55.6%)	8 (44.4%)

## Discussion

Surgery and chemotherapy remain the only treatments for MIBC; however, the post-surgery complication rate is high (up to 20%)^20^. Such complications may delay or preclude the use of adjuvant chemotherapy. CDDP is the first-line chemotherapeutic agent used for the treatment of advanced bladder cancer and is associated with an overall response rate of 33%^21^. For elderly and frail patients, especially patients who underwent radical cystectomy, the full course of CDDP chemotherapy is always difficult to endure. Oncolytic viral therapy has recently been recognized as a promising new therapeutic option for the treatment of cancer. In 2015, the U.S. Food and Drug Administration (FDA) approved the first oncolytic virus, Talimogene Laherparepvec (T-Vec, a genetically modified herpes simplex virus-1), for the treatment of malignancies^22^, this was a milestone of the research of oncolytic virus for the treatment of cancer. Our research demonstrated that the antitumor capacity of M1 for MIBC surpasses that of CDDP. In addition, we determined that low ZAP expression may be a biomarker for the usefulness of M1 precision therapy and was noted in 45.6% of 91 bladder cancer specimens. All of these results indicated that M1 has potential as a new agent in the treatment of advanced bladder cancer.

Thus far, only one oncolytic virus (Adenovirus CG0070) has been introduced in clinical trials for bladder cancer. The phase I trial showed that the intravesical delivery of virus reached complete response rates for single- and multi-dose regimens were 48.6% and 63.6%, respectively^23^, indicating that the CG0070 may be a promising agent in the treatment of nonmuscle-invasive bladder cancer. Intravenous systemic delivery may be more appropriate for MIBC. In our study, we delivered M1 intravenously and found that it exerted strong anticancer effects on orthotopic bladder tumors. These data indicate that M1 may be a good alternative treatment for bladder cancer.

Our data show that the killing activity of M1 differs among bladder cancer cell lines (Figure S1). Transcriptional profile analysis revealed that ISGs seem to be expressed at low levels in sensitive cancer cells compared with refractory cells, indicating that weaker antiviral signals are associated with greater M1 therapeutic efficacy. Consistent with these findings, previous studies have shown that ISG deficiency contributes to the susceptibility of cancer cells to oncolytic vesicular stomatitis virus^24^. ZAP, an ISG evaluated in our previous and current study, was found to be negatively correlated with the susceptibility of hepatoma, colon cancer and bladder cancer cells to M1 oncolytic therapy^18^. These correlations indicate that M1 may be useful as a precision oncolytic virotherapeutic agent. A companion diagnostic test measuring ZAP protein levels, which can serve as a predictive biomarker of M1 therapy responsiveness, may help doctors select those patients who are most likely to benefit from M1 therapy.

Our findings regarding the effects of ZAP knockdown or overexpression on M1 replication support the notion that ZAP acts as an anti-M1 factor in bladder cancer and are consistent with the findings of other studies regarding the antiviral role of ZAP in hepatitis B virus, human immunodeficiency virus-1^19,20^, etc. Additionally, it is important to note that gain of ZAP function in sensitive cells did not completely abrogate the losses in cell viability induced by M1 therapy and that loss of ZAP function did not induce a significant change in the killing efficacy of M1. These data imply that deficiencies in ISGs other than ZAP may also participate in rapid M1 replication in sensitive bladder cancer cells and that multiple ISGs may impede M1 invasion and infection in resistant cells. In this study, we determined that ZAP may be a predictor of the effectiveness of M1 oncolytic virotherapy. In the future, we plan to determine if other ISGs are strongly correlated with M1 antitumor activity and are thus useful as predictive biomarkers for the effectiveness of M1 precision therapy.

We noted low ZAP expression in 45.6% of patients who were believed to be suitable candidates for M1 oncolytic therapy. It is worth noting that low ZAP expression was common in clinically advanced MIBC (T3–4), indicating that M1 virotherapy be widely applicable for the treatment of advanced MIBC. Furthermore, we found that the poor prognosis of bladder cancer was closely related to ZAP deficiency, suggesting that low ZAP expression may be useful as a predictor of disease progression, prognosis, and survival in patients with advanced MIBC. Taken together, these findings indicate that ZAP may function as a tumor-suppressor gene affecting invasive bladder cancer development and progression. Coincidently, it has been reported that ZAP can promote tumor cell apoptosis in hepatoma by degrading TRAILR4 protein^25^. Thus, the exact role of ZAP deficiency in bladder cancer development requires further investigation.

In conclusion, our study showed that the oncolytic virus M1 selectively induces the apoptosis of bladder cancer cells without harming normal cells. The killing efficacy of M1 in bladder cancer cells is negatively correlated with ZAP expression, low expression of ZAP was observed in 45.6% of clinical bladder cancer specimen. Importantly, intravenous injections of M1 significantly repress orthotopic-invasive bladder cancer growth and greatly improve the survival of tumor-bearing mice—a finding supported by our observation that the weights of these mice did not change—to a greater extent than the first-line chemotherapy agent cisplatin. The potent therapeutic efficacy of M1 demonstrated herein, which is based on ZAP selectivity, indicates that the agent has promise as a precision therapy for bladder cancer, especially MIBC.

## Materials and methods

### Cell preparation and reagents

The cell lines used herein were purchased from the American Type Culture Collection and the Shanghai Institute of Cell Biology. The cells were cultured in Dulbecco’s modified Eagle’s medium (Gibco Life Technologies) supplemented with 10% (vol/vol) fetal bovine serum and 1% penicillin/streptomycin (Life Technologies) at 37 °C in a humidified 5% CO_2_ atmosphere.

Primary cultured bladder cancer cells were isolated from surgical tumor tissue samples obtained from consenting patients who underwent radical cystectomy. The human studies performed herein were approved by the Institutional Review Board of the Third Affiliated Hospital of Sun Yat-sen University. After extracorporealizing the bladder, we transferred the tumor tissues to a sterilized table, after which we dissociated primary bladder cancer cells from the body of the tumor using 0.1% trypsin, bladder tumor was diagnosed as urothelial carcinoma pathologically.

CDDP (S1166; 10 mg/ml, dissolved in dimethylformamide in vitro; 3 mg/ml dissolved in normal saline in vivo) was obtained from SelleckChemicals (Houston, TX).

### Virus storage

The M1 and GFP-labeled M1 used in this study were grown in Vero cells (OPTI- SFM, 12309-019, Thermo Fisher, Waltham, MA). Viral titers in BHK-21 cells were determined via TCID_50_ assay and converted to PFUs.

### Cell viability assay

Cells in the exponential growth phase were seeded in 24-well plates in 0.2 ml of media per well at a density of 30,000 cells per well. After 12 h, M1 (MOI = 10 PFU per cell) and CDDP (10 μM) were added to the wells, as described in the legends. Forty-eight hours later, cell metabolic activity was determined by MTT assay and converted into cell viability, the method was noted in the previous study^26^.

### Quantitative reverse transcription polymerase chain reaction

RNA was extracted using TRIzol reagent (Life Technologies), and reverse transcription was performed with 3 μg of total RNA using oligo(dT) and RevertAid Reverse Transcriptase (Thermo Scientific), according to the manufacturer’s instructions. Gene amplification was performed with SuperReal PreMix SYBR Green (TIANGEN) using an Applied Biosystems 7500 Fast Real-Time PCR System (Life Technologies), and the expression levels of all the genes were normalized to those of β-actin. Primers for each exon region of ZAP, NS1, and β-actin have been reported^26^.

### Orthotopic animal model

The animal studies performed herein were approved by the Animal Ethics and Welfare Committee of Sun Yat-sen University. Four-to 5-week-old female BALB/c-nu/nu mice were obtained and maintained in groups of five in cages and allowed access to water and food ad libitum.

General anesthetic was induced in the mice with inhaled isoflurane (3% for induction, 1–2% for maintenance) and maintained via nosecone. Sterile ophthalmic ointment was applied to the animal’s eyes, and a heat pad was used to maintain body heat. The animals were placed in the supine position, the abdominal wall of each mouse was sterilized with chlorhexidine, a low longitudinal laparotomy incision was placed, and the urinary bladder was extracorporalized. In all, 50-μl suspension of freshly harvested UM-UC-3 cells (2.5 × 10^6^ cells) was inoculated directly into the emptied bladder wall using a 30G needle. The incision was then closed with absorbable suture, and the catheter was removed. The mice were subsequently randomized into the three groups, treatment regime is described in the legend.

### Immunohistochemistry assay

After the animals were euthanized and their tumor-bearing bladders were harvested, we measured cleaved-caspase-3 (9664s, Cell Signaling Technology) and Ki-67 (9449s, Cell Signaling Technology) expression levels in the tumors, the detailed method have been published elsewhere^26^.

### Tissue microarray (TMA)

The TMAs used herein were purchased from Alenabio Biotech Co., Ltd and Shanghai Biochip Co., Ltd. Complete patient clinical data (TNM staging, overall survival, and pathology results) were provided with all tissue chips. The detailed information have been reported^26^.

### Statistical analysis

All statistical analyses were performed using SPSS 13.0 software (SPSS, IBM, Armonk, NY). Most comparisons were performed using Student’s *t*-test or one-way analysis of variance followed by Dunnett’s multiple post hoc test. Unless otherwise indicated, all the error bars indicate SDs. Bar charts show the mean ± SD of three independent experiments, unless otherwise indicated. Pearson correlation coefficient was used to calculate statistical dependence. A *p*-value below 0.05 was considered significant.

## Electronic supplementary material

Supplementary
