# Supplementary material for: Intravenous injections of the oncolytic virus M1 as a novel therapy for muscle-invasive bladder cancer
Source: Cell Death Dis. 2018 Feb 15;9(3):274. doi: 10.1038/s41419-018-0325-3 (PMC5833719; doi:10.1038/s41419-018-0325-3)
Supplement: Supplementary file 1 — Supplementary [file 41419_2018_325_MOESM1_ESM.docx]

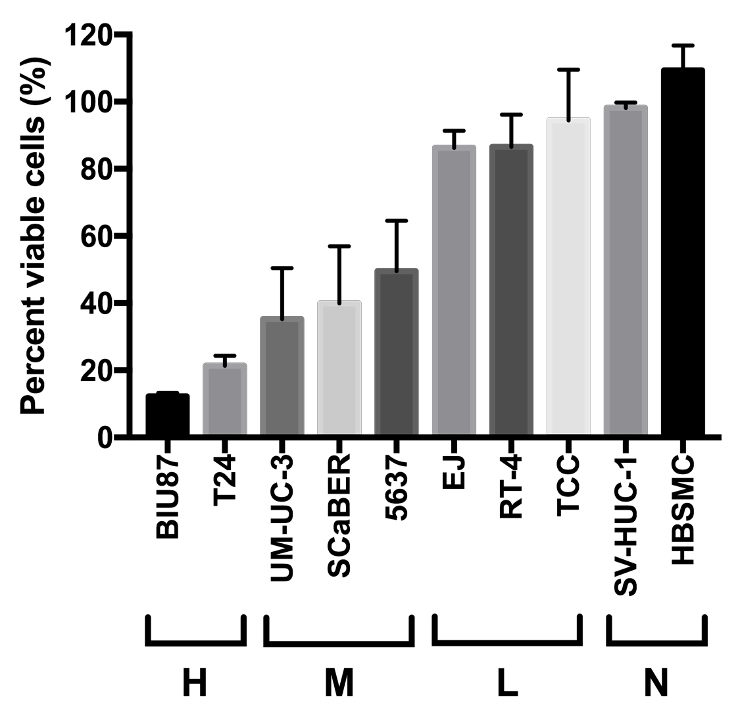


**Fig. S1. Sensitivity of bladder cancer cells to M1.**

Cells were infected with (MOI=10 PFU) M1, and cell viability was determined at 48 hours post-infection. The sensitivities of bladder cancer cells and normal cells to M1 were classified as follows: highly sensitive (H), moderately sensitive (M), and lowly sensitive (L). N normal cells.


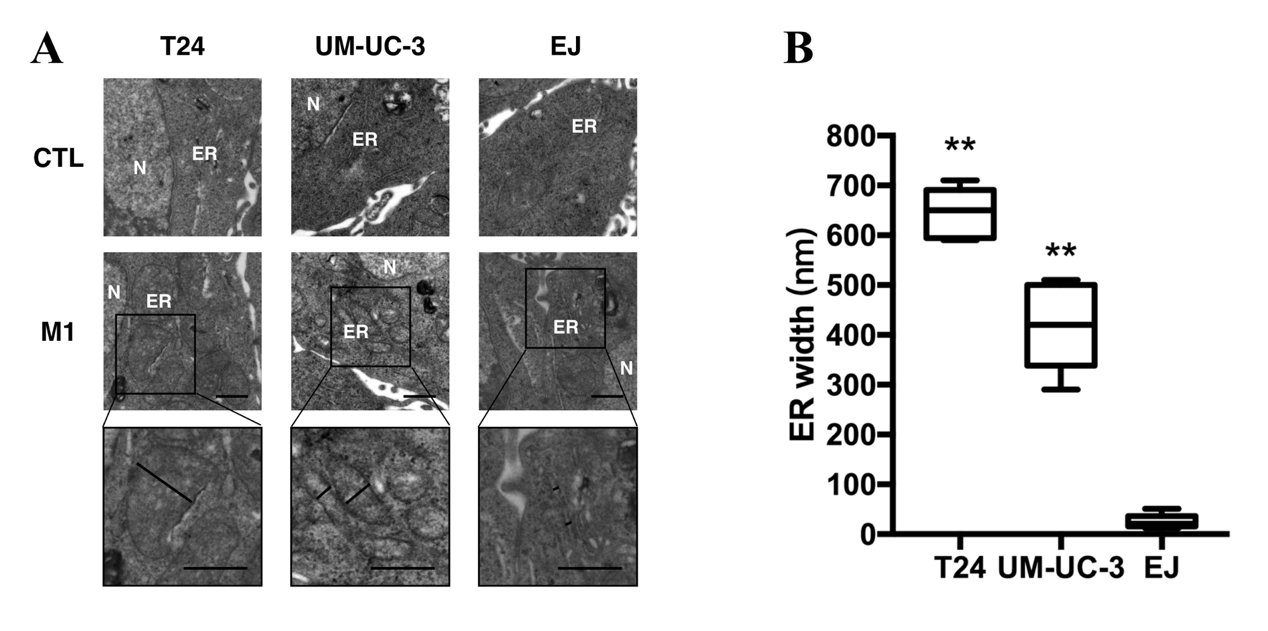


**Fig. S2. M1 causes ER stress in sensitive cancer cells.**

1. Transmission electron microscopy images of ER distention in highly sensitive T24 cancer cells, moderately sensitive UM-UC-3 cancer cells and lowly sensitive EJ cancer cells. The lower images are high-magnification images of the middle image (9,700, up and middle; 13,800, down, Scale bar: 500 nm). (B) Quantitative results pertaining to ER distension are also presented (mean ± SD). ***p*<0.01.


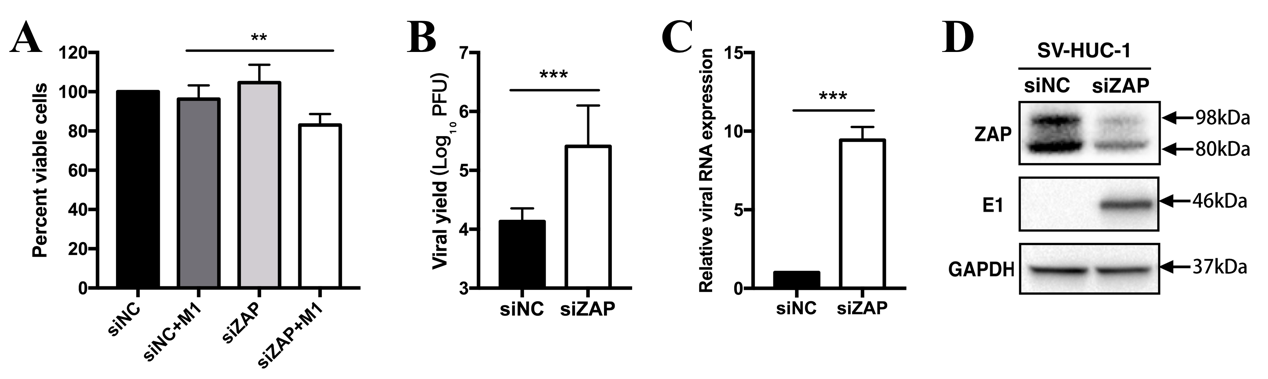


**Fig. S3. Knocking down ZAP in normal bladder cell SV-HUC-1 enhances the killing efficacy of M1.**

ZAP was silenced in normal cell line SV-HUC-1 with siNC (negative control) and siZAP, and then the cells were infected with M1 (MOI=10 PFU) for 48 hours. Cell viability was detected by MTT assay(A), viral replication levels were determined by TCID_50_ essay(B), viral RNA expression was quantified by qRT-PCR(C), and viral protein expression was analyzed by western blotting(D). ***p*<0.01; ****p*<0.001.
